# Occurrence and characteristics of *Escherichia coli mcr‐1*‐like in rabbits in Shandong, China

**DOI:** 10.1002/vms3.340

**Published:** 2020-10-03

**Authors:** Xinxing Wang, Zhenzhen Zhai, Xiaonan Zhao, Hongna Zhang, Hanming Jiang, Xuepeng Wang, Hairong Wang, Weishan Chang

**Affiliations:** ^1^ College of Animal Science and Technology Shandong Agricultural University Tai'an China; ^2^ School of Public Health Shandong First Medical University & Shandong Academy of Medical Sciences Tai'an China; ^3^ Postdoctoral Scientific Research Station Tai'an City Central Hospital Tai'an China; ^4^ Institute of Animal Science and Veterinary Medicine Shandong Academy of Agricultural Sciences Jinan China; ^5^ Department of Teaching Affairs Hebei University of Economics and Business Shijiazhuang China; ^6^ Department of Biochemistry School of Basic Medical Sciences Shandong First Medical University & Shandong Academy of Medical Sciences Tai'an China

**Keywords:** *Escherichia coli*, *mcr‐1*, plasmids, prevalence, rabbits

## Abstract

Polymyxin is regarded as the last retort to fight against multidrug‐resistant (MDR) *Enterobacteriaceae*. The emergency and spread of polymyxin‐associated resistance gene *mcr‐1* evoked great panic of no medicine to cure the bacterial infection in society. *mcr‐1* is widespread in domestic and wild animals. Therefore, continuous monitoring of its prevalence and characteristics is required. In this study, we used a polymerase chain reaction (PCR)‐based method to detect the *mcr‐1* of *Escherichia coli* isolated from rabbits of Tai'an, China, and determined the characteristics of *mcr‐1‐*bearing plasmids. A total of 55 non‐duplicated *E. coli* was recovered from the swabs of rabbit faeces. Plasmid profiling, plasmid and chromosome PCR, complete genome sequencing, a conjugation experiment, lactose fermentation experiment, multilocus sequence typing and polymyxin resistance tests were performed to determine the characteristics of *mcr‐1‐*bearing plasmids. 14.6% (8/55) of the specimens were *mcr‐1* positive. The *mcr‐1*‐positive *E. coli* harboured more drug‐resistant genes compared with the *mcr‐1*‐negative specimens, and results showed four sequence types. Overall, these findings suggested the possible threat of the transmission of *mcr‐1* from rabbits to humans, especially since the gene is located on transferable plasmids making horizontal transfer relatively easy. Since food‐producing animals are necessary for our daily diet, worldwide cooperation is needed in fighting the spread of this drug resistance gene to avoid human infections with MDR pathogenic bacteria.

## Graphical abstract

The *mcr‐1*‐positive *Escherichia coli* were more likely to be multidrug resistance, which makes it difficult to cure. The results also suggest the possible threat of the horizontal transfer of *mcr‐1* from rabbits to humans.

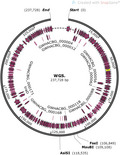

## 1 INTRODUCTION

In China, antibiotics were very commonly used in fighting against rabbit bacterial inflammation, which was the main problem in Chinese rabbit breeding. The use of antibiotics can disrupt the balance of normal microbial colonies in the cecum of rabbits, and cause bacterial diseases and digestive disorders in rabbits. *Escherichia coli* in China has antibiotic resistance to most types of genes and the most prevalent genes are ESBLs and quinolones. Both ESBLs and PMQR genes were detected in faecal *E. coli* isolated from the non‐human primates in six zoos in China, and the prevalence of ESBL‐encoding genes was 32%, and the prevalence of PMQR genes was 33% (Yang et al., 2012). In China, the output of rabbit meat is more than 400,000 tons, and the export volume is maintained between 20,000 tons and 30,000 tons every year (Agnoletti, Brunetta, Bano, Drigo, & Mazzolini, 2018). Colistin had been forbidden to use in human medicine for its nephrotoxicity, however, it was still useful in rabbits to prevent diseases like diarrhoea and promote growth, and drug‐resistant genes are now widely distributed in the intestines of farm animals, which are continuously being identified (Briñas et al., 2002; Naseer & Sundsfjord, 2011; Bryan, Shapir, & Sadowsky, 2004). The emergence of *mcr‐1*, a plasmid‐mediated colistin resistance gene, has alerted the public health systems and led to changes in how resistance is perceived globally. Liu firstly reported that *mcr‐1* widely existed in *E. coli*, which was the most common host of *mcr‐1* in China (Liu et al., 2016). Until now, *mcr‐1* gene has been identified in ten diversified species of the *Enterobacteriaceae*, isolated from over 40 countries/regions (Sun, Zhang, Liu, & Feng, 2018). Following this pattern, it is likely that drug‐resistant bacteria are present in rabbit faeces (Gao et al., 2015; Zhao, Ye, Chang, & Sun, 2017). Polymyxin was considered as a promising antimicrobial peptide, and very few bacteria showed polymyxin resistance. However, Chinese researchers identified *mcr‐1* as a gene conferring resistance to colistin and polymyxin (Liu et al., 2016). Although *mcr‐1* has been reported and detected worldwide, its global prevalence remains largely unknown. Liu et al. (2016) screened for *mcr‐1* in *E. coli* in raw pork and found that the gene was located on plasmids. The prevalence of *E. coli mcr‐1* in rabbits in China has not been reported. In these studies, the key methods to detect the location of genes were mainly based on Southern blotting. However, their detection methods were not based on polymerase chain reaction (PCR). Therefore, in this study, we employed a simpler method to determine the location and characteristics of *E. coli mcr‐1* among rabbits in China, that was, PCR combined with complete genome sequencing, which can help in estimating the existence, location and prevalence of *mcr‐1*. We also developed a method of combination of conjugation, PCR and fermentation test to further prove that plasmid harboured *mcr‐1*. For the final determination, we applied complete genome sequencing to the *mcr‐1*‐positive strains.

## 2 MATERIALS AND METHODS

### 2.1 Sample collection and identification of E. coli

The rabbits had been raised in large rabbit farms, free from thirst or starvation, and without signs of anxiety, fear or depression. The farms generally had 300~500 basic female rabbits. The formula for rabbit feed was 17% corn, 24% bran, 21% soybean meal, 5% imported fish meal, 3% active yeast and 30% grass powder. Meanwhile, polymyxin was added to feed with a proportion of 1:10,000. Faecal samples were randomly collected from the diarrhoea rabbits on three farms by rectal swabs, and the faeces were from individual animals. The three farms were separately selected in three administrative counties. Because the sampling process did not harm the rabbits, ethical approval was not required for the study.

Sixty rabbit faeces were collected in aseptic tubes (Agnoletti, 2012; Boullier & Milon, 2006), and plated on MacConkey agar to select and identify *E. coli*. The suspicious colonies were identified by bacterial biochemical tests. After biochemical identification, Gram stain and microscopic examination were performed to observe the morphology of the bacteria for confirmation. Positive colonies were then chosen for further biochemical identification using the automated API 20E system (Sysmex bioMérieux).

Recovered *E. coli* was cultivated in Luria‐Bertani liquid medium containing 2 μg/mL polymyxin B, and positive specimens were selected as the PCR detection templates.

### 2.2 PCR detection of mcr‐1

We further attempted to amplify *mcr‐1* from extracted plasmids and bacterial chromosomes. The samples of *mcr‐1*‐positive strains were separated and the plasmids were extracted with the OMEGA plasmid kit (Omega Bio‐Tek Co., Ltd.) and subjected to electrophoresis. The extracted plasmid was used as the template for PCR.

The DNA isolated with the method of alkaline lysis from *E. coli* strains was amplified by PCR using *mcr‐1*‐specific primers, F: 5ʹ AGTAGGCGTTTATTTGATAAATACGGCA 3ʹ; R: 5ʹ TTATATCAGATAAATTGTACTGGATTTC 3ʹ. PCR systems included 25 μL PCR mix, 21 μL deionized water, 1 μL forward primer, 1 μL reverse primer and 2 μL template, for a total of 50 μL (Shanghai Sangon Co., Ltd.). The reaction programme was as follows: 94°C pre‐denaturation for 5 min, followed by 28 cycles of 94°C denaturation for 30 s, annealing at 55°C for 30 s, and 72°C extension for 30 s. A final extension step was conducted at 72°C for 7 min. The PCR products of *mcr‐1* were then subjected to electrophoresis at 140 V for 30 min. The positive specimens were sent to Sangon for direct sequencing for confirmation (White, Mclver, Deng, & Rawlinson, 2006), and the sequences of *mcr‐1*‐positive strains were compared by the BLAST of the National Center for Biotechnology Information website.

PCR was then carried out with the extracted plasmid as a template using primers specific to *mcr‐1* and other resistance genes including *bla*_TEM_ under the same PCR conditions described earlier (Table S1). Similarly, bacterial genome chromosomes were extracted and purified from the samples of *mcr‐1*‐positive strains, and PCR analysis was performed with *mcr‐1*‐specific primers as described earlier.

### 2.3 Plasmid characterization and sequencing

One sample was randomly selected for sequencing. The concentration of the extracted genome was determined using the method of both Qubit Fluorometer and Nanodrop. Those meeting the requirements of sequencing were sent for sequencing, and the coding genes and structure were analysed by bioinformatics, such as spade, prokka and Pfam2go database. Through a comparative analysis of the extracted plasmids, the *E. coli* R45 strain carrying the *mcr‐1* gene was ultimately selected, and the extracted plasmid from this strain was designated pR45. A whole‐genome shotgun strategy was used to construct libraries of different inserted fragments. Paired‐end sequencing was performed on the Illumina MiSeq platform. SPAdes genome assembler (v 3.7.1) was used to construct contigs and the scaffold by the ab initio assembly of sequencing data, which were then removed and corrected.

### 2.4 Conjugation experiments

To prove that the antibiotic resistance gene in *E. coli* has the ability to transfer in vitro, 55 *mcr‐1*‐harbouring *E. coli* strains were isolated, which were resistant to polymyxin but sensitive to sodium azide. *E. coli* J53 was resistant to sodium azide and sensitive to most antibiotics. Conjugative testing was performed using the filter mating method, mixing at a ratio of 1:1 in broth culture, as previously described (Zhang, Zhou, Guo, & Chang, 2015). The transfer rate was determined, subsequently. The resulting transconjugants were selected on brain heart infusion agar plates supplemented with polymyxin B (2 mg/L) and sodium azide (Guardabassi, Schwarz, & Lloyd, 2004). The conjugated bacteria were also observed using plasmid extraction and electrophoresis analysis. Transfer of the resistance gene was considered to have taken place when the plasmids were transferred from the wild‐type *mcr‐1*‐positive bacterium to the recipient bacterium.

### 2.5 Antimicrobial susceptibility testing

The K‐B method was used to detect the sensitivity of the isolated strains to ciprofloxacin (CIP), chloramphenicol (C), nalidixic acid (NA), amoxicillin/clavulanic acid (AML), tobramycin (TB), ceftazidime (CAZ), ceftriaxone (CRO), gentamicin (GEN), sulphamethoxazole/tremethoprim (SXT), imipenem (IMP), tetracycline (TET), ampicillin (AMP), cefoxitin (FOX), polymyxinB (PB) and amikacin (AMK) (Hangzhou Binhe Microorganism Reagent Co., Ltd.). For this assessment, the *E. coli* strain ATCC25922 was used as the quality control strain (CLSI, 2013). *E. coli* isolates resistant to more than three classes of antimicrobials were defined as multidrug‐resistant (MDR) isolates ( Moawad et al., 2017).

### 2.6 Multilocus sequence typing

According to http://bigsdb.pasteur.fr/ecoli/primers_used.html, eight pairs of primers for housekeeping genes (*dinB, icdA, pabB*, *polB, putP, trpA, trpB* and *uidA*) were designed and used for PCR (Zhao, Yang, Ju, Chang, & Sun, 2018). The products of PCR amplification were then sequenced by Shanghai Sangon Biotech Co., Ltd. The results were amended using Chromas and DNAStar software and then submitted to the Pasteur online database for processing. The allele number of each housekeeping gene was obtained and the sequence type (ST) of each strain was acquired (Dotto, Giacomell, Grilli, Ferrazzi, & Carattoli, 2013).

### 2.7 Phylogenetic analysis

The phylogenetic tree of the eight *mcr‐1* sequences and *mcr‐1* genes on GeneBank was constructed by a maximum likelihood method using Megalign 7.1.0 (DNAstar Co., Ltd) to determine the relationships among strains.

## 3 RESULTS

### 3.1 Isolation and identification

A total of 55 *E. coli* strains were isolated from the overall 60 samples.

### 3.2 Prevalence of mcr‐1

**FIGURE 1:**
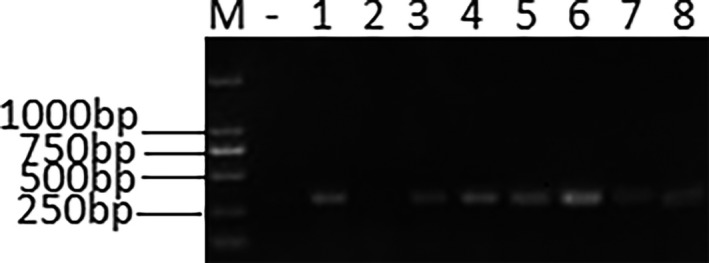
*mcr‐1*‐positive *Escherichia coli*

Eight specimens were found to be *mcr‐1* positive, representing a positive rate of 14.6% (Figure 1). The accession number of the eight sequences were MH395740, and MH602237‐MH602243. The *mcr‐1*‐positive strains harboured significantly more drug‐resistant genes other than *mcr‐1* compared to the *mcr‐1‐*negative strains (chi square test, *p* < .05) (Table 1). Thirteen different STs were identified among the 55 strains, with the most prevalent being ST302 (22/55, 40.0%), ST370 (12/55, 21.8%) and ST468 (5/55, 9.1%) (Table S2). Of note, the *mcr‐1*‐positive *E. coli* strains also showed a wide diversity of ST, although the dominant type was ST88 (62.5%).

**TABLE 1:** Comparison of multidrug‐resistant isolates detected in *mcr‐1‐*positive and ‐negative strains

	MDR	Rate	Non‐MDR	Rate
*mcr‐1* Positive	7	87.50%	1	12.50%
*mcr‐1* Negative	23	48.94%	24	51.06%

### 3.3 Plasmid sequencing results

The concentration of plasmid for analysis was 74.6 ng/μl. Complete genome sequencing was conducted on the *mcr‐1*‐positive strains. BLAST showed that *mcr‐1* was located on the plasmid. The extracted plasmid, designated pR45, encoding 19 predicted genes including *mcr‐1* (Table 2). The raw sequence data reported in this paper have been deposited in the Genome Sequence Archive, under accession number CRA002525 that is publicly accessible at https://bigd.big.ac.cn/gsa.

**TABLE 2:** Antibiotic resistance genes, resistance and resistance mechanism of pR45

Best_Hit_ARO	Resistance	Resistance mechanism
*AAC(3)‐IV*	Aminoglycoside antibiotic	Antibiotic inactivation
*OXA‐33*	Cephalosporin; penam	Antibiotic inactivation
*CTX‐M‐14*	Cephalosporin	Antibiotic inactivation
*mcr‐1*	Polymyxin	Antibiotic target alteration
*APH(3'')‐Ib*	Aminoglycoside antibiotic	Antibiotic inactivation
*tet(W/N/W)*	Tetracycline antibiotic	Antibiotic target protection
*FosA3*	Fosfomycin	Antibiotic inactivation
*AAC(6')‐Ib‐cr*	Aminoglycoside and fluoroquinolone antibiotic	Antibiotic inactivation
*sul2*	Sulphone and sulphonamide antibiotic	Antibiotic target replacement
*APH(6)‐Id*	Aminoglycoside antibiotic	Antibiotic inactivation
*tet(C)*	Tetracycline antibiotic	Antibiotic efflux
*APH(4)‐Ia*	Aminoglycoside antibiotic	Antibiotic inactivation
*floR*	Phenicol antibiotic	Antibiotic efflux
*arr‐3*	Rifamycin antibiotic	Antibiotic inactivation
*mphA*	Macrolide antibiotic	Antibiotic inactivation
*QnrS2*	Fluoroquinolone antibiotic	Antibiotic target protection
*catB3*	Phenicol antibiotic	Antibiotic inactivation
*Mrx*	Macrolide antibiotic	Antibiotic inactivation
*APH(3')‐Ia*	Aminoglycoside antibiotic	Antibiotic inactivation

### 3.4 Drug resistance test

**FIGURE 2:**
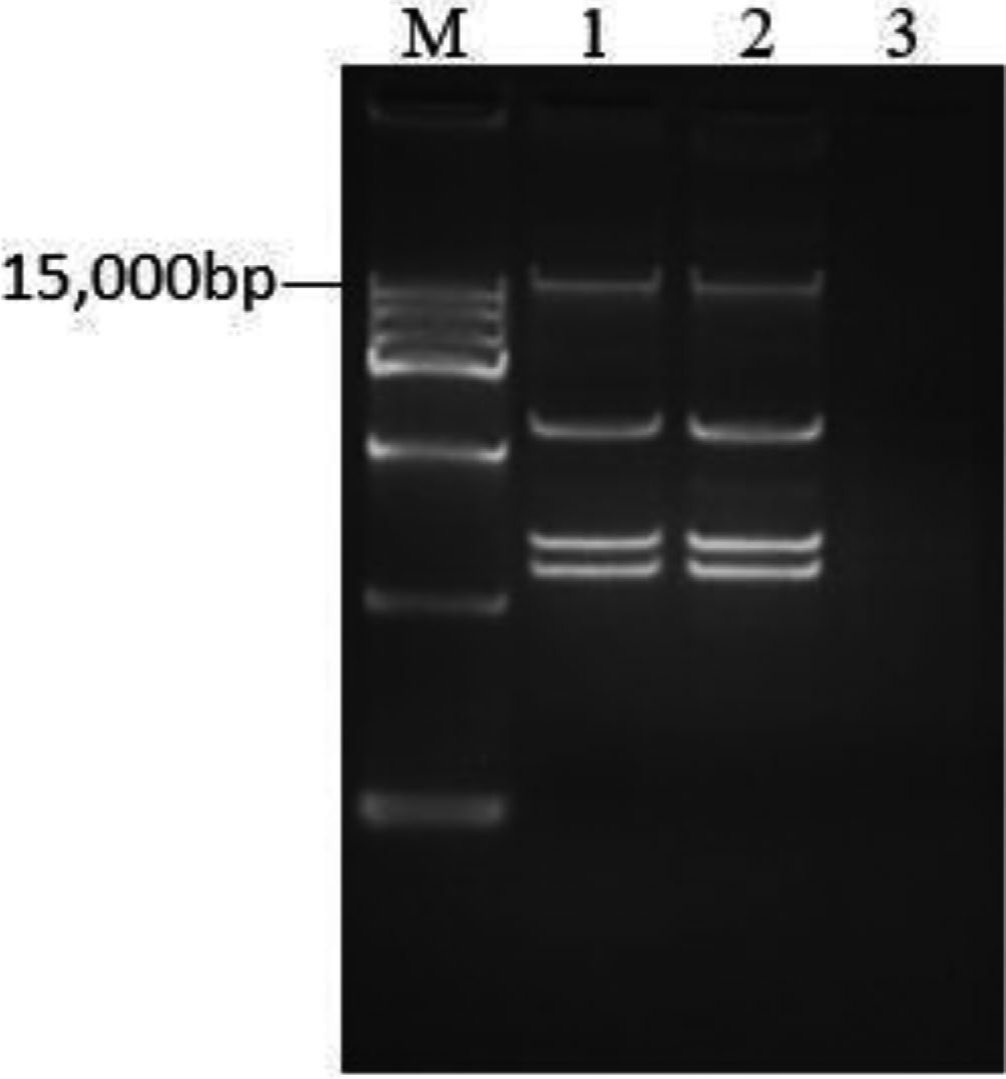
Plasmid profile of *mcr‐1*‐positive *Escherichia coli*. Lane 1 plasmid profile of donor, Lane 2 plasmid profile of recipient, Lane 3 negative control, M plasmid DNA marker

The results of drug resistance phenotyping and resistance were consistent with the results of *mcr‐1* detection. (Table 3). The results demonstrated that the *E. coli* resistance gene had transferability in vitro, and that the mobile plasmid played an important role in the process of drug resistance transmission in *E. coli*, among which ESBLs was included in this analysis as it is the most prevalent antimicrobial resistance genes in the samples (Table S2). Moreover, the plasmid profile of donor strain and recipient was the same, demonstrating that the plasmid of R45 transferred to J53 (Figure 2).

**TABLE 3:** Resistance phenotype, ST and resistance genes in *Escherichia coli* isolated from rabbit farms

No.	Location	ST	Resistance phenotype	Resistance
1	Xintai	ST88	AML‐AMP‐C‐CIP‐GEN‐ NA‐SXT‐TET‐**PB**	*bla*_CTX‐M_, *bla*_TEM_, *cmlA*, *flor*, *sul2*, *sul3*, *tetB*, ***mcr‐1***
2	Xintai	ST88	AMP‐C‐CIP‐GEN‐NA‐SXT‐TET‐**PB**	*bla*_CTX‐M_, *bla*_TEM_, *cmlA*, *flor*, *sul2*, *sul3*, *tetB*, ***mcr‐1***
3	Xintai	ST2	AMP‐C‐CIP‐NA‐SXT‐TET‐**PB**	*bla*_CTX‐M_, *bla*_TEM_, *cmlA*, *flor*, *sul3*, ***mcr‐1***
4	Xintai	ST88	AMP‐C‐CIP‐GEN‐NA‐SXT‐TET‐**PB**	*bla*_CTX‐M_, *bla*_TEM_, *cmlA*, *flor*, *sul2*, *sul3*, *tetB*, ***mcr‐1***
5	Xintai	ST353	C‐TET‐**PB**	*bla*_TEM_, *flor*, *qnrS*, *sul2*, ***mcr‐1***
6	Xintai	ST88	C‐CIP‐NA‐TET‐**PB**	*bla*_CTX‐M_, *bla*_TEM_, *flor*, *sul2*, *sul3*, *tetB*, ***mcr‐1***
7	Xintai	ST24	AML‐AMP‐TET‐**PB**	*bla*_CTX‐M_, *bla*_TEM_, *flor*, *sul1*, ***mcr‐1***
8	Xintai	ST88	AMP‐C‐CIP‐GEN‐NA‐SXT‐TB‐TET‐**PB**	*bla*_CTX‐M_, *bla*_TEM_, *cmlA*, *flor*, *sul2*, *sul3*, *tetB*, ***mcr‐1***

The bold words indicate the topic of this case study. PB is the drug that mcr‐1‐positive bacteria are resistant to. The article aimed to investigate the prevalence of mcr‐1. We highlight mcr‐1 and PB to illustrate the link between the drug resistant phenotype and .PB resistance gene mcr‐1.

## 4 Conjugation tests

**FIGURE 3:**
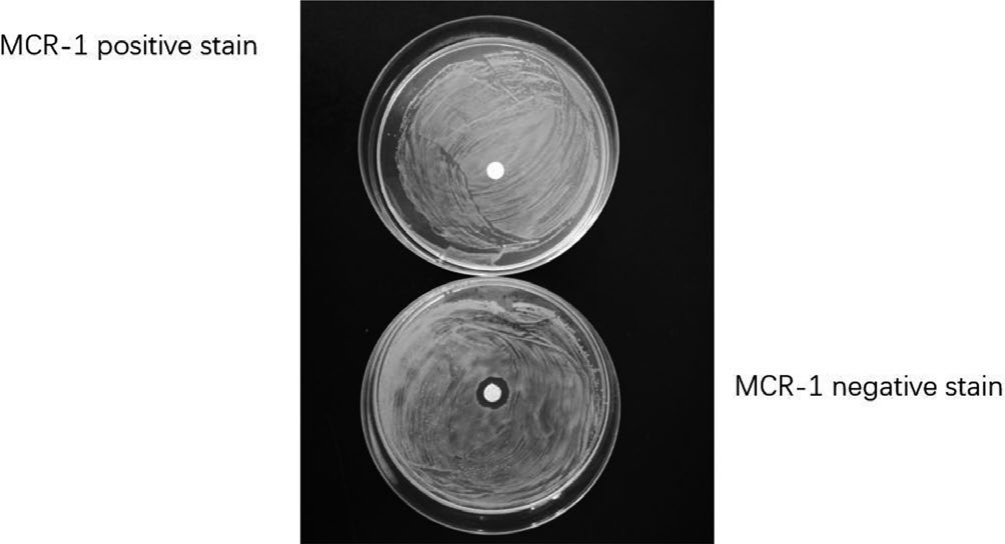
Drug sensitivity test

To prove the transferability of mobile plasmids in vitro, 55 strains of *E. coli* were used as donor bacteria, and 39 transconjugants were obtained successfully with the transfer rate as high as 70%. The conjugation tests confirmed the horizontal transfer of *mcr‐1* in *E. coli* strains obtained from rabbit faeces, therefore proving that *mcr‐1* was located on plasmids (Figure 3).

## 5 Phylogenetic analysis

**FIGURE 4:**
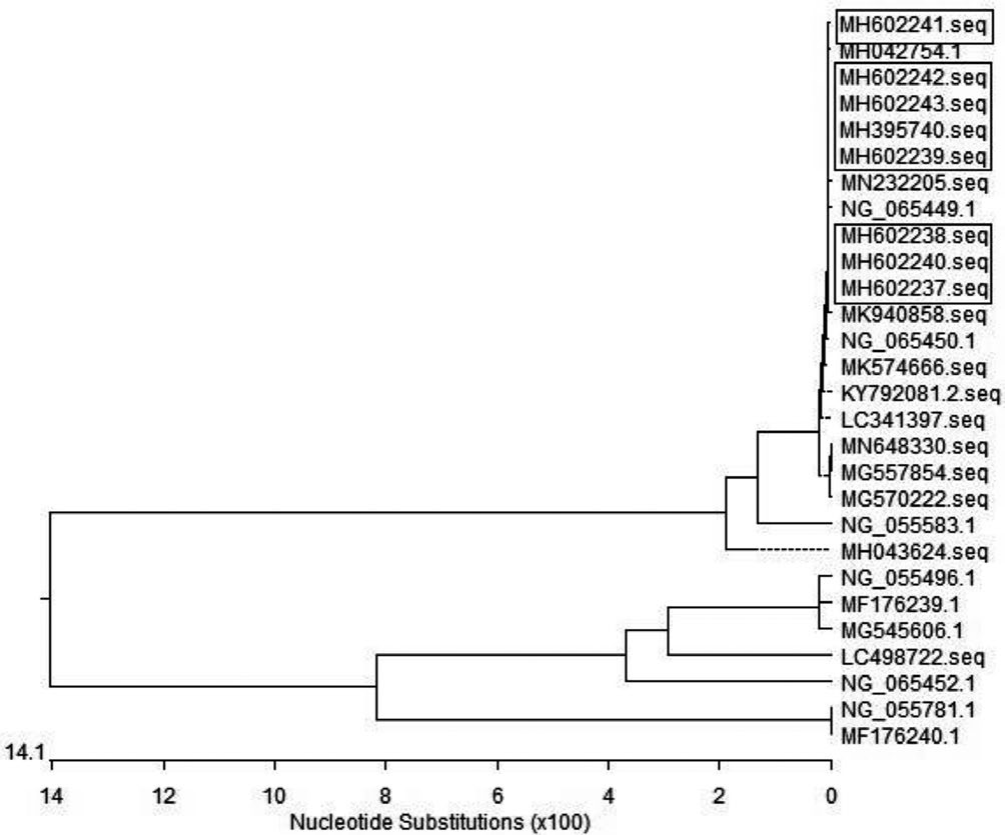
Phylogenetic tree of *mcr‐1* detected in *Escherichia coli* isolated from rabbits. The sequence of *Escherichia coli* from rabbits are in the square frame

Phylogenetic tree showed the evolutionary relationships among the eight *mcr‐1* sequences, demonstrating that although the eight positive strains were non‐duplicated *E. coli*, their *mcr‐1* sequences were identical (Figure 4).

## 6 Characteristics of mcr‐1

**FIGURE 5:**
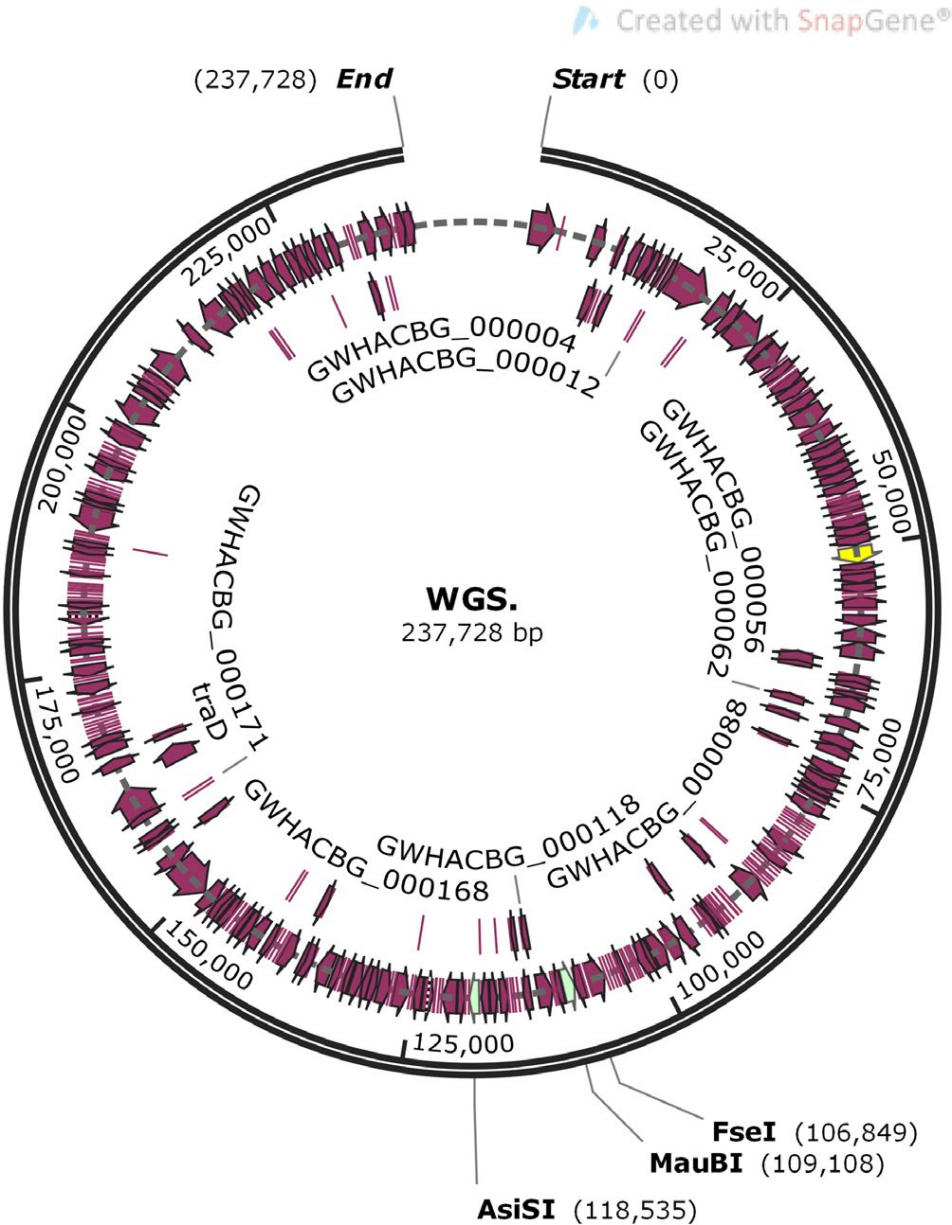
Structure of plasmid pR45 carrying *mcr‐1* from *Escherichia coli* strain R45. The part in yellow ranging from 49,992 to 51,617 bp is *mcr‐1*

Structure of plasmid pR45 showed that the whole length of the plasmid was 237,728 bp, and *mcr‐1* ranged from 49,992 to 51,617 bp (Figure 5). The structure of plasmid finally determined that *mcr‐1* located on the plasmid.

## 7 DISCUSSION

The prevalence of *mcr‐1* (8/55, 14.6%) detected in *E. coli* strains obtained from rabbits is similar to that reported in a study conducted in Italy (50/320, 15.6%) (Fabrizio, Romina, Luca, Ilenia, & Elena, 2018), and is markedly higher than that reported for humans (1~2%) (Yi, Liu, Wu, Liang, & Liu, 2017). This high rate may be due to the greater use of polymyxin in farms than in clinical practice. Most importantly, all of the *mcr‐1*‐positive strains obtained in this study were isolated from a single farm among the three sampled farms which was perhaps because the amount of polymyxin use varied across the different farms, which would impose different selection pressures on *mcr‐1*.

Because of the limitation of the total amount of specimens, it is difficult to generalize the results overall. Nevertheless, the antibiotic PCR tests demonstrated that the *mcr‐1*‐positive plasmids were more likely to harbour other resistant genes than *mcr‐1*‐negative plasmid. Accordingly, the *mcr‐1*‐positive *E. coli* had a greater probability of being MDR than *mcr‐1*‐negative *E. coli* (*p* < .05). Bacteria without plasmids readily gained donor bacterium plasmids and the *mcr‐1* gene along with the ability for lactose fermentation and polymyxin resistance at the same time. Therefore, these results strongly suggest the high horizontal dissemination potential of *mcr‐1*.

Moreover, the low diversity of *mcr‐1* sequences among the *E. coli* strains indicated that the *mcr‐1* gene was most likely derived from the same source, further suggesting clonal transmission of *E. coli* and horizontal transmission of *mcr‐1‐*harbouring plasmids in this area. This may be related to the fact that this region is relatively isolated, far from the city, with minimal flow of people.

Although *mcr‐1* gene was very conservative, they have diverse STs, demonstrating that *mcr‐1* had different origins. The resistance gene *mcr‐1* was found in eight strains of bacteria, which showed that the presence of plasmids for bacteria made it possible to produce drug resistance and survive in adversity. Resistance genes not only transfer from one bacterium to another or from one bacterium species to other species but also move geographically consequently (Kun et al., 2006). Therefore, the threat of drug resistance is not localized to a given animal farm or region, but represents a worldwide concern requiring global cooperation. Indeed, the fact that the bacterial resistant gene is located on the plasmid makes it potentially more difficult to control than a chromosomal gene. Plasmid transmission makes the spread of drug resistance genes easier and faster, and since the same plasmid can carry a variety of resistance genes, the recipient can immediately become resistant to multiple drugs. This finding suggested that it would be very difficult to cure humans infected with MDR pathogenic bacteria (Valat et al., 2016).

## 8 CONCLUSION

The conjugation test and complete genome sequence analysis of the ligated plasmid demonstrated that the *E. coli* resistance gene *mcr‐1* was circulating in rabbits of Eastern China, with the ability for horizontal transfer in vitro, indicating that the mobile plasmid played an important role in the process of antibiotic resistance of *E. coli* (Silva et al., 2010). As the antimicrobial resistance‐positive bacterial strains can survive in the presence of antibiotics, the bacteria can readily acquire additional drug resistance genes, resulting in a new MDR phenotype for the bacteria. Therefore, continuous selective pressure of antibiotics in farms will result in the production of new drug resistance genes that can readily circulate among domestic and wild animals, and even humans. To prevent the impact of *mcr‐1* on humans, we should first reduce the probability of *mcr‐1*‐harbouring strains in humans for proliferation and infection. Governments should carefully monitor and report the use of antibiotics in their jurisdictions. It is possible to effectively control the further spread of *mcr‐1* in humans and animals and to curb the development of polymyxin resistance. The whole world should cooperate to deal with the problem of drug resistance.

## CONFLICT OF INTEREST

The authors declare that the research was conducted in the absence of any commercial or financial relationships that could be construed as a potential conflict of interest.

## AUTHOR CONTRIBUTION

**Xinxing Wang:** Data curation; Formal analysis; Methodology; Visualization; Writing‐original draft. **Zhenzhen Zhai:** Formal analysis; Funding acquisition; Software; Validation; Visualization. **Xiaonan Zhao:** Formal analysis; Investigation; Software. **Hongna Zhang:** Validation. **Hanming Jiang:** Investigation; Supervision. **Xuepeng Wang:** Conceptualization; Funding acquisition; Project administration; Supervision. **Weishan Chang:** Conceptualization; Funding acquisition. **Hairong Wang:** Funding acquisition.

### PEER REVIEW

The peer review history for this article is available at https://publons.com/publon/10.1002/vms3.340.

## Supporting information

Supplementary Material
