# Supplementary material for: Occurrence and characteristics of Escherichia coli mcr‐1‐like in rabbits in Shandong, China
Source: Vet Med Sci. 2020 Oct 3;7(1):219–25. doi: 10.1002/vms3.340 (PMC7840214; doi:10.1002/vms3.340)
Supplement: Supplementary file 1 — Supplementary Material [file VMS3-7-219-s001.docx]

Supplementary Material

Occurrance and characteristics of *Escherichia coli* *mcr-1*-like in rabbits in China

Xinxing Wang, Zhenzhen Zhai, Xiaonan Zhao, Hongna Zhang, Hanming Jiang, Xuepeng Wang, Hairong Wang*, Weishan Chang*

*** Correspondence:**

Weishan Chang: [1651542031@qq.com](mailto:1651542031@qq.com)

Hairong Wang: [wanghairongtaian@1](mailto:xpwang@sdau.edu.cn)63.com

**Supplementary Table S1.** Primers used for PCR.

| Primer name | Sequence (5′→ 3′) |
| --- | --- |
| *mcr-1*  *bla*_TEM_ | F: CGGTCAGTCCGTTTGTTC  R:CTTGGTCGGTCTGTAGGG  F: ATTCTTGAAGACGAAAGGGC |
|  | R: ACGCTCAGTGGAACGAAAAC |
| *bla*_SHV_ | F: CACTCAAGGATGTATTGTG |
|  | R: TTAGCGTTGCCAGTGCTCG |
| *bla*_OXA_ | F: ACACAATACATATCAACTTCGC |
|  | R: AGTGTGTTTAGAATGGTGATC |
| *bla*_PSE_ | F: TTT GGT TCCGCG CTA TCT G |
|  | R: TAC TCC GAG CAC CAA ATC CG |
| *bla*_CTX-M_ | F: CGCTTTGCGATGTGCAG |
|  | R: ACCGCGATATCGTTGGT |
| *qnrA* | F: ATTTCTCACGCCAGGATTTG |
|  | R: TGCCAGGCACAGATCTTGAC |
| *qnrB* | F: CGACCTKAGCGGCACTGAAT |
|  | R: GAGCAACGAYGCCTGGTAGYTG |
| *qnrS* | F: ATGGAAACCTACAATCATAC |
|  | R: AAAAACACCTCGACTTAAGT |
| *aac(6’)-Ib-cr* | F: TTGCGATGCTCTATGAGTGGCTA |
|  | R: CTCGAATGCCTGGCGTGTTT |
| *qepA* | F: GCAGGTCCAGCAGCGGGTAG |
|  | R: CTTCCTGCCCGAGTATCGTG |
| *aac(3)-Ⅰ* | F: ACCTACTCCCAACATCAGCC |
|  | R: ATATAGATCTCACTACGCGC |
| *aac(3)-Ⅱ* | F: ACTGTGATGGGATACGCGTC |
|  | R: CTCCGTCAGCGTTTCAGCTA |
| *aac(3)-Ⅲ* | F: CACAAGAACGTGGTCCGCTA |
|  | R: AACAGGTAAGCATCCGCATC |
| *aac(3)-Ⅳ* | F: CTTCAGGATGGCAAGTTGGT |
|  | R: TCATCTCGTTCTCCGCTCAT |
| *ant(2")* | F: ATGTTACGCAGCAGGGCAGTCG |
|  | R: CGTCAGATCAATATCATCGTGC |
| *aac(6')-Ib* | F: TTGCGATGCTCTATGAGTGGCTA |
|  | R: CTCGAATGCCTGGCGTGTTT |
| *tetA* | F: GCGCCTTTCCTTTGGGTTCT |
|  | R: CCACCCGTTCCACGTTGTTA |
| *tetB* | F: CATTAATAGGCGCATCGCTG |
|  | R: TGAAGGTCATCGATAGCAGG |
| *sul1* | F: TGGTGACGGTGTTCGGCATTC |
|  | R: GCGAGGGTTTCCGAGAAGGTG |
| *sul2* | F: CGGCATCGTCAACATAACC |
|  | R: GTGTGCGGATGAAGTCAG |
| *sul3* | F: CATTCTAGAAAACAGTCGTAGTTCG |
|  | R: CATCTGCAGCTAACCTAGGGCTTTGGA |
| *cmlA* | F: TGTCATTTACGGCATACTCG |
|  | R: ATCAGGCATCCCATTCCCAT |
| *flor* | F: CTGAGGGTGTCGTCATCTAC |
|  | R: GCTCCGACAATGCTGACTAT |

**Supplementary Table S2. Characteristics of *mcr-1* of *E. coli* in rabbits.**

| No. | Location | ST | Resistance phenotype | Resistance |
| --- | --- | --- | --- | --- |
| 1 | Region 1 | ST302 | AMP-NA-TET | *bla*_CTX-M_, *bla*_TEM_, *sul2* |
| 2 | Region 1 | ST302 | AMP-TET | *bla*_CTX-M_, *bla*_TEM_, *sul2* |
| 3 | Region 1 | ST302 | AMP-NA-TET | *bla*_CTX-M_, *bla*_TEM_, *sul2* |
| 4 | Region 1 | ST302 | AMP-TET | *bla*_CTX-M_, *bla*_TEM_, *sul2* |
| 5 | Region 1 | ST468 | AMP-C-NA-SXT-TET | *bla*_CTX-M_, *bla*_TEM_,  *flor* |
| 6 | Region 1 | ST302 | AMP-CIP-NA-SXT-TET | *bla*_CTX-M_, *bla*_TEM_, *sul2* |
| 7 | Region 1 | ST468 | AMP-C-NA-SXT-TET | *bla*_CTX-M_, *bla*_TEM_, *sul2* |
| 8 | Region 1 | ST302 | AMP-TET | *bla*_CTX-M_, *bla*_TEM_, *sul2* |
| 9 | Region 1 | ST302 | AMP-TET | *bla*_CTX-M_, *bla*_TEM_, *sul2* |
| 10 | Region 1 | ST468 | AMP-CIP-NA-SXT-TET | *bla*_CTX-M_, *bla*_TEM_, *sul2* |
| 11 | Region 1 | ST302 | AMP-TET | *bla*_CTX-M_, *bla*_TEM_, *sul2* |
| 12 | Region 1 | ST370 |  | *bla*_CTX-M_, *bla*_TEM_ |
| 13 | Region 1 | ST370 |  | *bla*_CTX-M_, *bla*_TEM_, *sul1* |
| 14 | Region 1 | ST302 | AMP-TET | *bla*_CTX-M_, *bla*_TEM_, *sul1*, *sul2* |
| 15 | Region 1 | ST370 |  | *bla*_CTX-M_, *bla*_TEM_ |
| 16 | Region 1 | ST302 | AMP-NA-TET | *bla*_CTX-M_, *bla*_TEM_, *sul1*, *sul2* |
| 17 | Region 1 | ST302 | AMP-TET | *bla*_CTX-M_, *bla*_TEM_, *sul2* |
| 18 | Region 1 | ST370 |  | *bla*_CTX-M_, *bla*_TEM_ |
| 19 | Region 2 | ST370 | SXT | *bla*_CTX-M_, *bla*_TEM_, *sul1* |
| 20 | Region 2 | ST87 | GEN-SXT | *bla*_CTX-M_, *bla*_TEM_, *qnrS*, *sul1* |
| 21 | Region 2 | ST302 | AMP-GEN-SXT-TET | *bla*_CTX-M_, *bla*_TEM_, *sul2* |
| 22 | Region 2 | ST302 | AMP-NA-TET | *bla*_CTX-M_, *bla*_TEM_, *sul2* |
| 23 | Region 2 | ST370 |  | *bla*_CTX-M_, *bla*_TEM_ |
| 24 | Region 2 | ST302 |  | *bla*_CTX-M_, *bla*_TEM_, *sul1* |
| 25 | Region 2 | ST314 | NA-TET | *bla*_CTX-M_, *bla*_TEM_, *sul1*, *sul2* |
| 26 | Region 2 | ST302 | AMP-C-NA-SXT-TET | *bla*_CTX-M_, *bla*_TEM_, *flor*, *sul2* |
| 27 | Region 2 | ST302 | AMP-FOX-NA-TET | *bla*_CTX-M_, *bla*_TEM_, *sul2* |
| 28 | Region 2 | ST302 | TET | *bla*_CTX-M_, *bla*_TEM_, *sul2* |
| 29 | Region 2 | ST302 | AMP-NA-SXT | *bla*_CTX-M_, *bla*_TEM_, *sul2* |
| 30 | Region 2 | ST636 |  | *bla*_CTX-M_, *bla*_TEM_, *sul1* |
| 31 | Region 2 | ST370 | AMP-NA-TET | *bla*_CTX-M_, *bla*_TEM_, *sul2* |
| 32 | Region 2 | ST468 | AMP-NA-TET | *bla*_CTX-M_, *bla*_TEM_, *sul2* |
| 33 | Region 2 | ST468 | AMP-C-NA-SXT | *bla*_CTX-M_, *bla*_TEM_, *flor* |
| 34 | Region 2 | ST302 | AMP-NA-TET | *bla*_CTX-M_, *bla*_TEM_, *sul2* |
| 35 | Region 2 | ST302 | AMP-NA-TET | *bla*_CTX-M_, *bla*_TEM_, *sul2* |
| 36 | Region 2 | ST302 | AMP-NA-TET | *bla*_CTX-M_, *bla*_TEM_, *sul2* |
| 37 | Region 2 | ST302 | AMP -TET | *bla*_CTX-M_, *bla*_TEM_, *sul2* |
| 38 | Region 2 | ST370 | NA-TET | *bla*_CTX-M_, *bla*_TEM_, *sul1* |
| 39 | Region 3 | ST461 | AMP-NA-SXT | *bla*_TEM_, *qnrS*, *sul1* |
| 40 | Region 3 | ST731 | TET | *bla*_TEM_ |
| 41 | Region 3 | ST739 | AMP-NA-TET | *acc(6’)-Ib-cr*, *bla*_CTX-M_, *bla*_TEM_ |
| 42 | Region 3 | ST370 | TET | *bla*_CTX-M_, *bla*_TEM_ |
| 43 | Region 3 | ST739 | AMP-CIP- NA-SXT-TET | *acc(6’)-Ib-cr*, *bla*_CTX-M_, *bla*_TEM_ |
| 44 | Region 3 | ST370 | TET | *bla*_CTX-M_, *bla*_TEM_ |
| 45 | Region 3 | ST88 | AML-AMP-C-CIP-GEN- NA-SXT-TET-**PB** | *bla*_CTX-M_, *bla*_TEM_, *cmlA*, *flor*, *sul2*, *sul3*, *tetB,* ***mcr-1*** |
| 46 | Region 3 | ST370 | TET | *bla*_CTX-M_, *bla*_TEM_, *sul1* |
| 47 | Region 3 | ST739 | AMP-CIP-NA-SXT-TET | *acc(6’)-Ib-cr*, *bla*_CTX-M_, *bla*_TEM_ |
| 48 | Region 3 | ST88 | AMP-C-CIP-GEN-NA-SXT-TET-**PB** | *bla*_CTX-M_, *bla*_TEM_，*cmlA*, *flor*, *sul2*, *sul3*, *tetB,* ***mcr-1*** |
| 49 | Region 3 | ST2 | AMP-C-CIP-NA-SXT-TET-**PB** | *bla*_CTX-M_, *bla*_TEM_, *cmlA*, *flor*, *sul3,* ***mcr-1*** |
| 50 | Region 3 | ST88 | AMP-C-CIP-GEN-NA-SXT-TET-**PB** | *bla*_CTX-M_, *bla*_TEM_, *cmlA*, *flor*, *sul2*, *sul3*, *tetB,* ***mcr-1*** |
| 51 | Region 3 | ST353 | C-TET-**PB** | *bla*_TEM_, *flor*, *qnrS*, *sul2,* ***mcr-1*** |
| 52 | Region 3 | ST370 | NA-TET | *bla*_CTX-M_, *sul1* |
| 53 | Region 3 | ST88 | C-CIP-NA-TET-**PB** | *bla*_CTX-M_, *bla*_TEM_, *flor*, *sul2*, *sul3*, *tetB,* ***mcr-1*** |
| 54 | Region 3 | ST24 | AML-AMP-TET-**PB** | *bla*_CTX-M_, *bla*_TEM_, *flor*, *sul1,* ***mcr-1*** |
| 55 | Region 3 | ST88 | AMP-C-CIP-GEN-NA-SXT-TB-TET-**PB** | *bla*_CTX-M_, *bla*_TEM_, *cmlA*, *flor*, *sul2*, *sul3*, *tetB,* ***mcr-1*** |
